# Copy number variation in Williams-Beuren syndrome: suitable diagnostic strategy for developing countries

**DOI:** 10.1186/1756-0500-5-13

**Published:** 2012-01-09

**Authors:** Roberta L Dutra, Rachel S Honjo, Leslie D Kulikowski, Fernanda M Fonseca, Patrícia C Pieri, Fernanda S Jehee, Debora R Bertola, Chong A Kim

**Affiliations:** 1 Department of Genetics, Instituto da Criança, Universidade de São Paulo, São Paulo, Brazil; 2 Department of Pathology, LIM 03, Universidade de São Paulo, São Paulo, Brazil; 3 Laboratory of Genomic Pediatrics - LIM 36, Instituto da Criança, Universidade de São Paulo, São Paulo, Brazil

## Abstract

**Background:**

Williams-Beuren syndrome (WBS; OMIM 194050) is caused by a hemizygous contiguous gene microdeletion at 7q11.23. Supravalvular aortic stenosis (SVAS), mental retardation, and overfriendliness comprise typical symptoms of WBS. Although fluorescence in situ hybridization (FISH) is considered the gold standard technique, the microsatellite DNA markers and multiplex ligation-dependent probe amplification (MLPA) could be used for to confirm the diagnosis of WBS.

**Results:**

We have evaluated a total cohort of 88 patients with a suspicion clinical diagnosis of WBS using a collection of five markers (D7S1870, D7S489, D7S613, D7S2476, and D7S489_A) and a commercial MLPA kit (P029). The microdeletion was present in 64 (72.7%) patients and absent in 24 (27.3%) patients. The parental origin of deletion was maternal in 36 of 64 patients (56.3%) paternal in 28 of 64 patients (43.7%). The deletion size was 1.55 Mb in 57 of 64 patients (89.1%) and 1.84 Mb in 7 of 64 patients (10.9%). The results were concordant using both techniques, except for four patients whose microsatellite markers were uninformative. There were no clinical differences in relation to either the size or parental origin of the deletion.

**Conclusion:**

MLPA was considered a faster and more economical method in a single assay, whereas the microsatellite markers could determine both the size and parental origin of the deletion in WBS. The microsatellite marker and MLPA techniques are effective in deletion detection in WBS, and both methods provide a useful diagnostic strategy mainly for developing countries.

## Background

Williams-Beuren syndrome (WBS; OMIM 194050) is a neurodevelopmental disorder described independently [1,2] as a syndrome involving facial appearance characteristics, supravalvular aortic stenosis (SVAS) and mental retardation. In fact, WBS presents a wide collection of symptoms affecting blood vessels, growth, intelligence, and behavior. Children with this condition have distinctive facial features, a hoarse voice associated with growth, mental retardation and an overfriendly personality; hyperacusis, infantile hypercalcemia, prematurely wrinkled skin are also common symptoms [3].

WBS is generally sporadic with frequency of approximately 1 in 7,500 live births with no ethnic or sex preference, although familial cases have been reported with apparent autosomal dominant inheritance [4,5]. Despite the consistency of the overall clinical features, the broad spectrum of anomalies and phenotypic variability frequently lead to a significant difference in the number of patients diagnosed [6].

WBS is caused by a hemizygous contiguous gene microdeletion of the WBS critical region on chromosome 7 at position 7q11.23. The most common deletion is found in 90% to 95% of WBS patients and spans a genomic region of approximately 1.55 Mb. It is the result of mispairing between the centromeric and medial LCR (Low copy repeats) blocks B (Bcen and Bmid) [7]. In 5% to 10% of cases, the breakpoints are within the centromeric and medial LCR blocks A (Acen and Amid) and lead to an ~1.84-Mb deletion [8]. Atypical (approximately 0.2 Mb to ~2.5 Mb) deletions may be the leading cause of the substantial phenotypic variability among WBS patients [9].

Duplication of the WBS region occurs at half the frequency of deletions with less distinctive and somehow opposite clinical features, such as deficits of social interaction and an autistic-like phenotype [10,11].

Confirmation of clinical suspicion is essential for clinical monitoring of the patient and genetic counseling of the family. Although fluorescence in situ hybridization (FISH) is widely used and considered the gold standard for WBS molecular diagnosis, the use of microsatellite DNA markers has also been widely used and is considered highly informative and easily performed [12,13].

Multiplex ligation-dependent probe amplification (MLPA) has been introduced into DNA diagnostic laboratories for the detection of deletions and/or duplications in several disease genes [14]. MLPA kit for WBS, makes possible a more precise mapping of the deletion in the critical region, compared with the FISH [15]. In this study, the results obtained with microsatellite markers were compared with those obtained with MLPA.

It can be argued that both techniques, together, are extremely valuable tools for the diagnosis of the WBS patients and that the implementation of both methods should be considered.

## Results

A total of 88 patients with the suspicion of a clinical diagnosis of WBS were tested. The five markers (D7S1870, D7S489, D7S613, D7S2476 and D7S489A) were informative in 84 patients and not informative in 4 patients. The most informative marker was D7S1870 (78.4% of patients), followed by D7S613 (68.2% of patients), D7S489 (65.9% of patients) and D7S2476 (57.9% of patients). The microdeletion was present in 64 (72.7%) patients and absent in 24 (27.3%) patients.

The observed deletion size was 1.55 Mb in 57 of 64 patients (89.1%) and 1.84 Mb in 7 of 64 patients (10.9%). For the parental origin, the deletion was maternal in 36 of 64 patients (56.3%) and paternal in 28 of 64 patients (43.7%).

**Figure 1:**
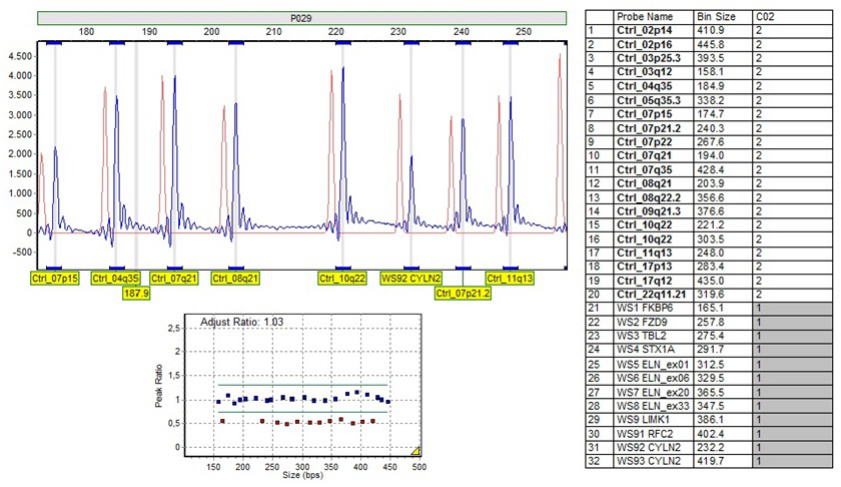
**Genotyping by MLPA technique (SALSA kit P029) using the software GeneMarker^® ^for analysis**. Hemizygous contiguous gene microdeletion, can be visualized by probes 21 to 32. With the presence one copy these genes in the WBS critical region, 7q11.23.

Using the MLPA kit (P029), the results were concordant with the microsatellite marker analysis in 84 patients and on 4 cases the deletion was only detected by MLPA (Figure 1). FISH was performed in all patients and the results were concordant with those found by microsatellites and MLPA.

**Figure 2:**
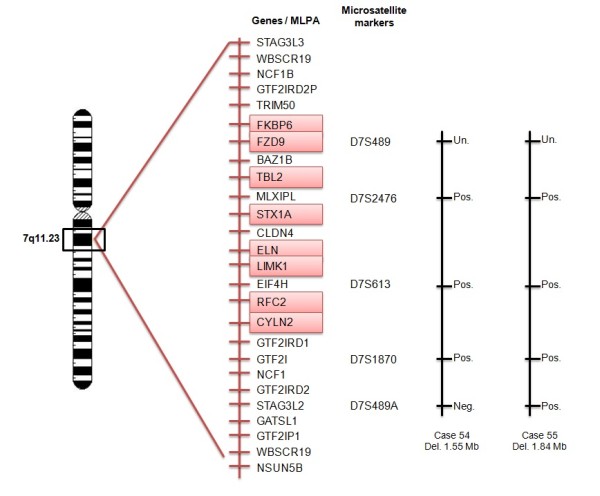
**Representation of the 7q11.23 region and the location of the probes from SALSA kit P029 and the markers tested**. The patients 54 and 55 were represented in the figure to illustrate the localization of the genes in the region 7q11.23 and the size of the deletion. Neg. Negative; Pos. Positive and Un. Uninformative.

**Figure 3:**
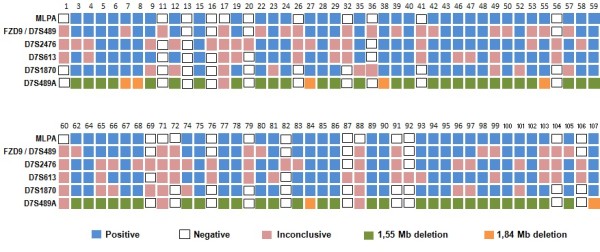
**Comparison between the results obtained by microsatellite markers and MLPA**. A total of 88 patients participated of the study and numbered 1 to 107. The correspondent probe to gene FZD9 is localized in the same region that D7S489 microsatellite marker.

The microsatellite markers used in the present study, are located in different regions in comparison with the probes in the P029 kit for WBS (Figure 2). Except the D7S489 marker and the FZD9 probe from MLPA P029 kit that are in the same position (Figure 3). Considering both techniques, there was no clinical difference in relation to either the size of deletion or the parental origin of deletion.

## Discussion

Microsatellite DNA markers and MLPA have been considered highly informative and easily manageable for diagnostic confirmation of WBS.

In our study, five microsatellite markers (D7S1870, D7S489, D7S613, D7S2476, and D7S489A) were informative, except in four cases.

However, in these particular cases, short stature, microcephaly, and cardiovascular anomalies were absent, but not in one patient that presented mitral and tricuspid regurgitation and hiperacusis.

The D7S1870 microsatellite marker showed the highest power of detection, able to identify 78.4% of the cases by itself, which confirmed the results from previous studies [8,16-19].

Two best markers (D7S1870 and D7S613) in our study were able to detect the deletion in 93.2% of cases when used together. When the D7S613 and D7S489 markers were included, informative detection increased to almost 95%.

The microsatellite marker D7S489A was effective in the analysis of deletion size. The 1.55-Mb deletion was found in 57 of 64 (89.1%) patients, and the 1.84-Mb deletion was found in 7 of 64 patients (10.9%); these observed percentages are similar to those found in other studies in the literature [8].

Using markers to identify the parental origin, we found no significant difference between the frequencies of maternal and paternal deletions (56.3% and 43.7%, respectively), and the literature is concordant with our findings [12,13,17,19].

There was also no relationship between clinical features with the size of the deletion and with the parental origin.

Since the MLPA technique was developed [14], it has been tested as a diagnostic method in several diseases involving chromosomal disorders. In this study, we used the MLPA kit (P029) to observe the microdeletion in 64 (72.7%) patients and find it was absent in 24 (27.3%) patients.

We find four discrepant results comparing the microsatellite markers and the MLPA method in the detection of deletions in the WBS critical region. In these patients where the microsatellite markers are uninformative, detection of the deletion can be confirmed using the MLPA technique. These patients present a phenotypic variability that often leads to diagnostic difficulties and the confirmation of results only was possible using MLPA technique.

The microsatellite markers were efficient in deletion detection for WBS when compared to the MLPA. They allowed for the detection of deletions larger than 1.55 MB and for detection of the parental origin of the deletion.

FISH is widely used and considered the gold standard for WBS molecular diagnosis, however, FISH is labor-intensive, time-consuming, and it does not allow the detection of the exact size of the deletion [20].

The cost of the microsatellite marker technique has greatly decreased, and it can be deployed in molecular biology laboratories that have basic equipment for conventional PCR reactions and a vertical electrophoresis system.

The most important advantages of the MLPA are its relative simplicity, low cost, rapid turnaround (2 days), ease of multiplexing to permit high confidence in the results, high accuracy of copy number estimation, and the potential for combination of copy number analysis with other applications, such as methylation detection or SNP genotyping [21].

The accuracy of both diagnostic tests is well recognized to be susceptible to technical problems and clinical heterogeneity. In our study, FISH, markers and MLPA presented higher sensitivity (99.8%), similar to others studies [22] and microsatellites markers presents lower specificity compared to FISH and MLPA (93%).

Real-time quantitative polymerase chain reaction technique (QPCR) and array-based comparative genomic hybridisation (array-CGH) are also being used for the molecular diagnosis of WBS.

QPCR is considered a robust methodology, with easy interpretation, and simple to set up [23,24]. Conversely, to perform this technique we need sophisticated equipments and specific primers for each target region, differently from MLPA, where the simultaneous hybridization of more than 40 different probes can be used in one single reaction.

Recently, array-CGH has also been proved also to be a powerful and promising method to detect microdeletions and to identify novel cytogenetic abnormalities [25]. However, the resolution of array-CGH can vary depending on the format and design of the array [26]. Additionally, this method is relatively difficult and costly, and it requires a different setup as far as instrumentation is concerned [25].

Economic models are important to help health professionals to take decisions based on available strategies. The molecular tests available together with socio economic characteristics of the country is fundamental when a new strategy is considered to be taken, especially in developing countries where resources are limited [27].

## Conclusions

The diagnosis of WBS based on clinical assessments may be difficult because of the great variability of its manifestations. Laboratory tests to detect the microdeletions in 7q11.23 are essential to confirm the clinical diagnosis of WBS.

In summary, the microsatellite marker and MLPA techniques are effective in deletion detection in WBS, and both methods improve complete molecular coverage in screening of the critical region mainly for developing countries.

## Methods

### Subjects

A total cohort of 88 patients with a clinical diagnosis of WBS (56 boys and 32 girls) were followed through clinical evaluation by geneticists of the Unit of Clinical Genetics - Instituto da Criança, Hospital das Clínicas - Universidade de São Paulo (ICr-HCFMUSP), Brazil. The inclusion criteria were dysmorphic facial features suggestive of WBS and the presence of cardiovascular disorders, mainly SVAS.

The study was approved by the Institutional Review Board - Ethics Committee for Analysis of Research Projects HCFMUSP/Cappesq - and written consent was obtained from all participants.

Among the 88 patients, DNA from both parents was obtained in 80 cases; in 8 cases, the molecular analysis was performed only with maternal DNA. Most of patients had normal GTG band karyotype and FISH was previously had been done in 24 patients.

The molecular study was performed in Laboratory of Genomic Pediatrics - LIM 36 - (Icr -HCFMUSP). DNA was isolated from peripheral blood lymphocytes using a salt precipitation technique [28].

### Microsatellite markers

The five microsatellites markers used included D7S1870, D7S489, D7S613 and D7S2476 inside the common 1.55-Mb deletion and D7S489A to distinguish deletions of 1.84 Mb. PCR reactions were carried out according to Dutra et al. (2011) [13].

Patient genotypes were compared with those of their parents. Deletions were diagnosed as maternal when the proband presented with gel bands representing the allelic marker inherited only from the father. When by chance both parents have the same alleles, the monoallelic inheritance of the corresponding microsatellite marker by the proband indicated an uninformative result.

We first used a two-step algorithm to identify the most common 1.55-Mb deletion. We then tested the D7S489A marker either to identify the larger 1.84-Mb deletion (in those patients in which a deletion of at least one marker was detected in the first step) or to confirm the lack of a deletion.

### MLPA

The MLPA (SALSA kit P029 - MRC-Holland, Amsterdam, The Netherlands) containing probes for eight genes from the WBS critical region (*FKBP6, FZD9, TBL2, STX1A, ELN, LIMK1, RFC2 *and *CYLN2*) were used. The *ELN *and *CYLN2 *probes for various exons are present in the kit. Denaturation, overnight hybridisation, ligation and PCR were performed according to the manufacturer's instructions.

MLPA products were separated on a MegaBACE™ 1000 (GE Life Sciences, Waltham, USA) using MegaBACE ET SIZE Standards ET550-R (GE Life Sciences, Waltham, USA). The analysis was performed using the GeneMarker, version 1.6, software (Softgenetics, State College, PA, USA). The ratio of the probes' peak heights was determined by comparing the probes' peak heights obtained from patient samples to those obtained from three normal control samples.

### Statistical analysis

Pairwise comparisons between clinical features of WBS and the presence of deletion, clinical features and deletion size and clinical features and parental origin of deletion were tested for significance using two-tailed Fisher's exact test. A 2 × 2 contingency table was used to compare clinical features. P analysis was performed in SPSS 13.0 software and considered statistically significant when p ≤ 0.05.

## Competing interests

Non-financial competing interests.

## Authors' contributions

RLD graduate student (PhD), involved in drafting the manuscript, participated in the design of the study and collaborated with analysis and interpretation of data. RSH Medical and graduate student (PhD) participated in the design of the study and collaborated with updating medical records and ambulatory care of Williams syndrome patients. LDK Biologist PhD, sponsor of Cytogenomics group, participated in the design of the study and collaborated with final review of the manuscript. FMF Technical laboratory LIM 36, collaborated with the microsatellites markers experiments. PCP Biologist in LIM 36, collaborated with the standardization of methods (microsatellite markers) and with interpretation of data. FSJ Biologist PhD, collaborated with the standardization of methods (MLPA) and with analysis and interpretation of data. DRB Assistant Doctor in Unit of Genetics, Instituto da Criança, FMUSP, collaborated with the ambulatory care of Williams syndrome patients. CAK Sponsor of the research of this work and responsible for Unit of Genetics, Instituto da Criança, FMUSP and participated with revising it critically for important intellectual content. All authors read and approved the final manuscript.

## References

[B1] Williams JC Barratt-Boyes BG Lowe JB Supravalvular aortic stenosis Circulation 1961 24 1311 1318 14007182 10.1161/01.cir.24.6.1311

[B2] Beuren AJ Apitz J Harmjanz D Supravalvular aortic stenosis in association with mental retardation and a certain facial appearance Circulation 1962 26 1235 1240 13967885 10.1161/01.cir.26.6.1235

[B3] Morris CA Demsey SA Leonard CO Dilts C Blackburn BL Natural history of Williams syndrome: physical characteristics J Pediatr 1988 113 318 326 10.1016/S0022-3476(88)80272-5 2456379

[B4] Strømme P Bjørnstad PG Ramstad K Prevalence estimation of Williams syndrome J Child Neurol 2002 17 269 271 10.1177/088307380201700406 12088082

[B5] Morris CA Thomas IT Greenberg F Williams syndrome: autosomal dominant inheritance Am J Med Genet 1993 47 478 481 10.1002/ajmg.1320470409 8256809

[B6] Ashkenas J Williams syndrome starts making sense Am J Hum Genet 1996 59 756 761 8808588 PMC1914814

[B7] Peoples R Franke Y Wang YK Pérez-Jurado L Paperna T Cisco M Francke U A physical map, including a BAC/PAC clone contig, of the Williams-Beuren syndrome--deletion region at 7q11.23 Am J Hum Genet 2000 66 47 68 10.1086/302722 10631136 PMC1288354

[B8] Bayés M Magano LF Rivera N Flores R Pérez Jurado LA Mutational mechanisms of Williams-Beuren syndrome deletions Am J Hum Genet 2003 73 131 151 10.1086/376565 12796854 PMC1180575

[B9] Gagliardi C Bonaglia MC Selicorni A Borgatti R Giorda R Unusual cognitive and behavioural profile in a Williams syndrome patient with atypical 7q11.23 deletion J Med Genet 2003 40 526 530 10.1136/jmg.40.7.526 12843326 PMC1735517

[B10] Berg JS Brunetti-Pierri N Peters SU Kang SH Fong CT Salamone J Freedenberg D Hannig VL Prock LA Miller DT Speech delay and autism spectrum behaviors are frequently associated with duplication of the 7q11.23 Williams-Beuren syndrome region Genet Med 2007 9 427 441 10.1097/GIM.0b013e3180986192 17666889

[B11] Van der Aa N Rooms L Vandeweyer G van den Ende J Reyniers E Fichera M Romano C Delle Chiaie B Mortier G Menten B Fourteen new cases contribute to the characterization of the 7q11.23 microduplication syndrome Eur J Med Genet 2009 52 94 100 10.1016/j.ejmg.2009.02.006 19249392

[B12] Sbruzzi IC Pereira AC Vasconcelos B Honjo RS Krieger JE Kim CA Williams-Beuren syndrome: diagnosis by polymorphic markers Genet Test Mol Biomarkers 2010 14 209 214 10.1089/gtmb.2009.0120 20136526

[B13] Dutra RL Pieri PC Teixeira AC Honjo RS Bertola DR Kim CA Detection of deletion at 7q11.23 in Williams-Beuren syndrome by polymorphic markers Clinics (Sao Paulo) 2011 66 6 959 964 10.1590/S1807-59322011000600007 PMC3129970 21808859

[B14] Schouten JP McElgunn CJ Waaijer R Zwijnenburg D Diepvens F Pals G Relative quantification of 40 nucleic acid sequences by multiplex ligation-dependent probe amplification Nucleic Acids Res 2002 30 e57 10.1093/nar/gnf056 12060695 PMC117299

[B15] van Hagen JM Eussen HJ van Schooten R van Der Geest JN Lagers-van Haselen GC Wouters CH De Zeeuw CI Gille JJ Comparing two diagnostic laboratory tests for Williams syndrome: fluorescent in situ hybridization versus multiplex ligation-dependent probe amplification Genet Test 2007 11 321 327 10.1089/gte.2007.0007 17949295

[B16] Karmiloff-Smith A Grant J Ewing S Carette MJ Metcalfe K Donnai D Read AP Tassabehji M Using case study comparisons to explore genotype-phenotype correlations in Williams-Beuren syndrome J Med Genet 2003 40 136 140 10.1136/jmg.40.2.136 12566524 PMC1735363

[B17] Pérez Jurado LA Peoples R Kaplan P Hamel BC Francke U Molecular definition of the chromosome 7 deletion in Williams syndrome and parent-of-origin effects on growth Am J Hum Genet 1996 59 781 792 8808592 PMC1914804

[B18] Gilbert-Dussardier B Bonneau D Gigarel N Le Merrer M Bonnet D Philip N Serville F Verloes A Rossi A Aymé S A novel microsatellite DNA marker at locus D7S1870 detects hemizygosity in 75% of patients with Williams syndrome Am J Hum Genet 1995 56 542 544 7847392 PMC1801136

[B19] Brøndum-Nielsen K Beck B Gyftodimou J Hørlyk H Liljenberg U Petersen MB Pedersen W Petersen MB Sand A Skovby F Investigation of deletions at 7q11.23 in 44 patients referred for Williams-Beuren syndrome, using FISH and four DNA polymorphisms Hum Genet 1997 99 56 61 9003495 10.1007/s004390050311

[B20] Merla G Brunetti-Pierri N Micale L Fusco C Copy number variants at Williams-Beuren syndrome 7q11.23 region Hum Genet 2010 128 1 3 26 10.1007/s00439-010-0827-2 20437059

[B21] Kozlowski P Jasinska AJ Kwiatkowski DJ New applications and developments in the use of multiplex ligation-dependent probe amplification Electrophoresis 2008 29 4627 4636 10.1002/elps.200800126 19053154

[B22] Bishop B Applications of fluorescence in situ hybridization (FISH) in detecting genetic aberrations of medical significance Biohorizons 2010 3 85 95

[B23] Howald C Merla G Digilio MC Amenta S Lyle R Deutsch S Choudhury U Bottani A Antonarakis SE Fryssira H Dallapiccola B Reymond A Two high throughput technologies to detect segmental aneuploidies identify new Williams-Beuren syndrome patients with atypical deletions J Med Genet 2006 43 266 273 15994861 10.1136/jmg.2005.034009 PMC2563253

[B24] Schubert C Laccone F Williams-Beuren syndrome: determination of deletion size using quantitative real-time PCR Int J Mol Med 2006 18 799 806 17016608

[B25] Snijders AM Nowak N Segraves R Blackwood S Brown N Conroy J Hamilton G Hindle AK Huey B Kimura K Assembly of microarrays for genome-wide measurement of DNA copy number Nat Genet 2011 29 263 264 10.1038/ng754 11687795

[B26] Shaffer LG Bejjani BA A cytogeneticist's perspective on genomic microarrays Hum Reprod Update 2004 10 221 226 10.1093/humupd/dmh022 15140869

[B27] Jehee FS Takamori JT Medeiros PF Pordeus AC Latini FR Bertola DR Kim CA Passos-Bueno MR Using a combination of MLPA kits to detect chromosomal imbalances in patients with multiple congenital anomalies and mental retardation is a valuable choice for developing countries Eur J Med Genet 2011 54 4 e425 e432 10.1016/j.ejmg.2011.03.007 21457803

[B28] Miller SA Dykes DD Polesky HF A simple salting out procedure for extracting DNA from human nucleated cells Nucleic Acids Res 1988 16 1215 10.1093/nar/16.3.1215 3344216 PMC334765

